# Evaluation of Remote Pharmacist-Led Outpatient Service for Geriatric Patients on Rivaroxaban for Nonvalvular Atrial Fibrillation During the COVID-19 Pandemic

**DOI:** 10.3389/fphar.2020.01275

**Published:** 2020-08-21

**Authors:** Xiaoye Li, Chengchun Zuo, Wenjing Lu, Ye Zou, Qing Xu, Xiaoyu Li, Qianzhou Lv

**Affiliations:** Department of Pharmacy, Zhongshan Hospital, Fudan University, Shanghai, China

**Keywords:** rivaroxaban, nonvalvular atrial fibrillation, corona virus disease 2019, remote medication management, pharmacist-led education, follow-up service

## Abstract

**Objective:**

This study was designed to evaluate the efficacy of remote medication management of rivaroxaban by pharmacists for geriatric patients with nonvalvular atrial fibrillation during the COVID-19 pandemic.

**Methods:**

A single-site, prospective cohort study was conducted among patients with non-valvular atrial fibrillation who received rivaroxaban therapy from July 2019 to December 2019. Patients in the pharmacist-led education and follow-up service (PEFS) group were managed remotely by a pharmacist. In contrast, those in the usual care (UC) group were managed by other providers. Data of routine blood tests, coagulation function tests, which also included cardiac function parameters were collected. The number and type of provider encounters, interventions related to rivaroxaban therapy, the occurrence of thromboembolism or bleeding, and the time of the first outpatient visit after discharge were recorded.

**Results:**

A total of 600 patients were recruited, and results of 381 patients were analyzed in the end, of which 179 patients were from the PEFS group and 202 were from the UC group. There was no significant difference between the two groups in terms of the occurrence ratio of systemic thrombosis, heart failure (LVEF < 40%), and left atrial dilation, which was defined as enlargement of left atrial diameter (LAD) > 40 mm. The cumulative incidences of bleeding complications, such as gastrointestinal tract and skin ecchymosis, were significantly higher in the UC group (12.4% vs. 6.1%, P=0.038; 4.5% vs. 0.6%, P=0.018). There was no significant difference after pharmacist intervention in terms of thrombosis occurrence ratio between the two groups (P = 0.338, HR: 0.722, 95% CI: 0.372-1.405). Remote instruction by a pharmacist reduced outpatient service frequency within the first 30 days after discharge (23.7% vs. 1.1%, P < 0.001). However, more patients in the PEFS group presented for the first outpatient revisit later than 40 days post-discharge (12.8% vs. 21.3%, P < 0.001).

**Conclusion:**

Remote pharmacist-led medication instruction of rivaroxaban could reduce bleeding complications of the gastrointestinal tract and skin ecchymosis and postpone the first outpatient revisit after discharge.

## Introduction

Corona Virus Disease 2019 (COVID-19), firstly confirmed in Wuhan, has drawn many concerns worldwide. COVID-19, which resembles the Middle East Respiratory Syndrome (MERS) and the Severe Acute Respiratory Syndrome (SARS), can cause acute respiratory infectious disease with clinical symptoms, such as fever, dry cough, and fatigue. In severe cases, patients may rapidly progress to acute respiratory distress syndrome, septic shock, and multiple organ failure (Valencia, 2020). Since January 2020, COVID-19 had spread in China and led to tremendous challenges to medical institutions (Li et al., 2020). To reduce the risk of cross-infection, medical institutions are currently advocating for a non-contact medical treatment model in China (Qian et al., 2020). The pharmaceutical department of the Zhongshan Hospital has built an efficient pharmaceutical service model that is convenient for patients through remote communication methods such as WeChat or telephone, to improve the service quality, reduce the risk of infection, and ensure the safety of patients.

Nonvalvular atrial fibrillation (NVAF) refers to atrial fibrillations without a mechanical or biological valve, rheumatic mitral stenosis, or history of mitral valve repair, which accounts for the vast majority of patients with NVAF (January et al., 2014). Epidemiological studies indicated that stroke incidence in patients associated with nonvalvular atrial fibrillation increases with age and the incidence of stroke in people aged 80-89 is about 36.2%(Pickett, 2019). The high risk of adverse drug events associated with the geriatric population was mainly due to irregular anticoagulation enforcement and insufficient medication self-management ability (Turner et al., 2012; Shore et al., 2014; Mielke et al., 2020).

Although under current COVID-19 pandemic hospital visits are limited and inconvenient, it is still vital to provide timely and personalized pharmaceutical care for geriatric patients with NVAF who are medicated with anticoagulant agents (Kim et al., 2019). Based on the above rationale, our study aimed to apply WeChat or telephone communication to provide remote pharmaceutical instruction and popularize the education of anticoagulants to enhance the efficacy and safety of rivaroxaban medication in geriatric patients during the pandemic.

## Methods

### Patients Information

This prospective single-site cohort study enrolled 600 participants from the Department of Cardiology, Zhongshan Hospital, Fudan University, from July 2019 to December 2019 following the protocols of the European Society of Cardiology (ESC) guideline (Kirchhof et al., 2016). All the patients were newly presented with a typical pattern of atrial fibrillation (AF) in the electrocardiogram (ECG) reading with absolutely irregular RR intervals and no discernible or distinct P waves. Integrated care was applied for all patients with these newly diagnosed AF including anticoagulation therapy after stroke risk assessment, rate and rhythm control therapy, and inconsistent approaches to cardiovascular risk reduction. All drugs were prescribed as single doses by nurses and pharmacists. For follow-ups, ECG and transthoracic echocardiography were performed during outpatient clinic visits by specialized cardiologists or other certified specialists. ECG monitoring in AF patients was suitable for the evaluation of the adequacy of heart rate control, related symptoms of AF recurrences, and detection of focal induction of bouts of paroxysmal AF. Transthoracic echocardiography was applied to assess left ventricular size and function (systolic and diastolic), atrial volume, right heart function, and identify structural diseases such as valvular disease.

As described above, all patients involved met the following eligibility criteria: 1) age ≥65 years; 2) high risk for stroke, transient ischemic attack, and systemic embolism in the CHA_2_DS_2_-VASc score (≥1); 3) received anticoagulation therapy rivaroxaban for at least one month. The main exclusion criteria included the following: 1) history of bleeding and hemorrhagic diseases; 2) prescribed with another anticoagulant; 3) severe renal dysfunction; 4) unable to communicate *via* WeChat or phone.

This study was conducted in compliance with the Helsinki Declaration and Good Clinical Practice and was approved by the Ethics Committee of Zhongshan Hospital. Written informed consent was signed by all participants before the commencement of the clinical studies.

### Pharmacist Care for Anticoagulation

Patients were eligible if they met the above criteria and started rivaroxaban with the intended duration of at least 3 months. Consecutive participants were allocated to receive either the pharmacist-led education and follow-up service (PEFS) or usual care (UC) with a ratio of 1:1 according to patients’ demand and willingness.

PEFS mainly consisted of rivaroxaban administration, observation of potential interaction between rivaroxaban and other drugs, and management of embolic complications and its related adverse reactions during anticoagulation treatment. The pharmaceutical education materials (Turpie, 2014) were listed in **Table 1**. On the first day of the study, paper-based medication education materials were distributed to patients, and telephone numbers or WeChat information were recorded. During the follow-up, clinical pharmacists focused on the evaluation of patient medication adherence, which was assessed by the doses missed on average and how/when the medication was taken. Pharmacists kept in touch with the patients through WeChat or telephone weekly and recorded the symptoms, including embolism, bleeding, or other adverse reactions during anticoagulation.

**Table 1:** Rivaroxaban Medication Guide.

**1. Reason for the use of Rivaroxaban in nonvalvular atrial fibrillation**		
□Patients with atrial fibrillation are prone to thrombosis which will cause stroke and other embolic diseases. Rivaroxaban is a direct Factor Xa inhibitor, which can reduce thrombin production and inhibit thrombosis.		
**2. Basic Information**		
General Name: Rivaroxaban	Specification: 20 mg/15 mg/10 mg	Dosage Form: Tablet
**3. Use**		
□ Please follow the doctor’s advice. Take medicine at the same time every day. Cutting tablets is allowed under the doctor’s order. □ Rivaroxaban (15 mg/20 mg) should be taken with meals. Rivaroxaban (10 mg) can be taken without meals.		
**4. Medication Monitor**		
□In general, the routine monitoring of coagulation indicator is not needed. □In special cases, such as suspected overdose, emergency surgery, severe bleeding event, need for thrombolysis, prothrombin time (PT) can be measured to assess the risk of rivaroxaban bleeding.		
**5. Circumstances that Rivaroxaban is forbidden (contraindication)**		
£ Allergic to any excipients in rivaroxaban or tablets; £ Active bleeding; £ Abnormal clotting and bleeding-risk liver damage, including Child-Pugh B and Cirrhosis C; £ Ccr < 15 mL/min; PLT < 20×10^9^/L.		
**6. Adverse reactions and corresponding preventive measures to Rivaroxaban**		
£Bleeding symptoms: Minor bleeding includes nosebleeds, gum bleeding, ecchymosis, etc. First, the drug should be delayed or suspended, and local compression should be used to stop bleeding. Severe bleeding includes gastrointestinal bleeding, gross hematuria, etc; life-threatening bleeding includes intracranial bleeding, medical advice should be sought immediately. □**Serious **thrombus symptoms: When symptoms appear, including Limb numbness, headaches of unknown cause, dyspnea of unknown cause, **medical advice should be sought immediately**.		
**7. Drug effects on Rivaroxaban**		
□Drugs that increase anticoagulation: HIV Protease Inhibitor, Azole antifungals (Itraconazole, Voriconazole, Posaconazole); □Drugs that reduce anticoagulation: Rifampicin, Phenobarbital, Phenytoin, Carbamazepine; □A combination of nonsteroidal anti-inflammatory drugs (such as Naproxen, Ibuprofen) may increase the risk of bleeding risk.		
**8. Other Attentions**		
□Please replenish Rivaroxaban within 4 hours after forgetting to take the medicine. Please do not take Rivaroxaban after 4 hours, continue to take the medicine the next day; □Drug overdose: Patients without bleeding should take activated carbon in a short time after taking Rivaroxaban or go to the hospital for gastric lavage. Patients with bleeding should take measures for bleeding symptoms.		

The UC was defined as a control group with routine medication education after discharge. Patients enrolled in the UC group received no education or guidance from a pharmacist during anticoagulation. The UC providers included cardiologists or primary care providers, depending on who was primarily responsible for the management of patient anticoagulation therapy.

### Data Collection

Demographic information was collected from electronic medical records (EMR), including age, gender, weight, date of admission/discharge, daily rivaroxaban medication, concomitant medication during hospitalization, and cardiovascular risk factors, such as hypertension, myocardial infarction, hyperuricemia, hyperlipidemia, renal insufficiency. All tests were conducted in the Department of Medical Laboratory in our hospital, which is International Organization for Standardization (ISO) 15189 certified. An estimated Glomerular Filtration Rate (eGFR) of < 60 mL/(min·1.73 m^2^) was classified as renal dysfunction, and the eGFR was calculated based on the modification of diet in renal disease (MDRD) equation. Routine coagulation parameters (activated partial thromboplastin time, prothrombin time, fibrinogen, and platelet), blood glucose parameters (glycosylated hemoglobin, fasting glucose, postprandial glucose), liver function parameters (bilirubin, binding bilirubin, albumin, globulin), and cardiac biomarkers were recorded. The embolism and bleeding risk score was evaluated according to ESC guidelines (Kirchhof et al., 2016).

### Clinical Outcomes

Routine blood tests, coagulation function tests (prothrombin time, activated partial thromboplastin time, fibrinogen, D-dimer value), and concomitant medication were recorded. Various indicators of cardiac structure and function under cardiac ultrasound were evaluated during the follow-ups. The clinical endpoint was major adverse cardiovascular events (MACE), including ischemic stroke, cardiovascular death, cardiovascular mortality, and recurrence of atrial fibrillation. The safety endpoint was defined as bleeding complications, such as hematuria, epidural hematoma, operation site hemorrhage, and bleeding gums. The number of visits and the time between discharge and the first visit were recorded.

### Statistical Analysis

The numerical data are presented as mean ± standard deviation (SD). Categorical variables are expressed with frequencies ± percentage. Independent t-test was used in parametric data, and the Mann-Whitney U test was used in nonparametric data. The baseline characteristics were compared between the PEFS and UC groups. Results of the anticoagulation effect for rivaroxaban were compared by t-test. The comparison of hemoglobin (Hb) and platelet (PLT) levels changes and bleeding complications were analyzed by t-test. We analyzed MACE using multiple statistical models. Variables including the occurrence of irregular RR intervals with no discernible or distinct P waves, left ventricular ejection fraction (LVEF), and left atrial dilation were analyzed. Hazard ratios (HRs) with two-sided 95% confidence intervals (CIs) were calculated for the risk factors of MACE. Results are presented as HRs along with 95% CI. The number of visits and the time between discharge and the first visit were constructed using Kaplan-Meier estimates. A two-sided P value was used to determine significance (threshold, P < 0.05). Statistical analysis was performed using SPSS (IBM SPSS Statistics 22.0) and Prism 5 (GrandPad Software). A P value of 0.05 was considered to be the threshold for statistical significance.

## Results

### Recruitment Process

**Figure 1:**
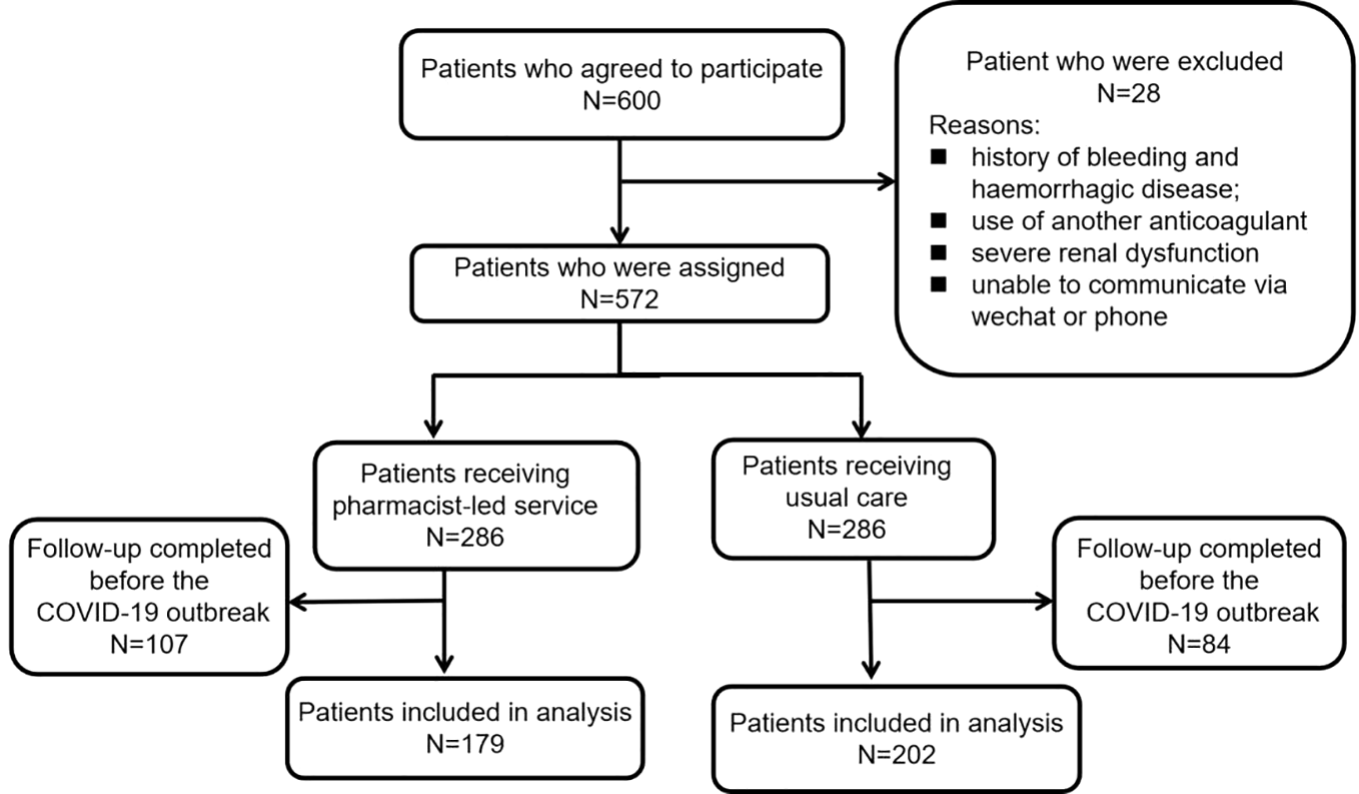
Flowchart recruitment procedure of the study.

During the recruitment period, 600 eligible patients consented to participate in this study. The recruitment procedure of the study and the reasons for exclusion are presented in **Figure 1**. 28 patients were excluded for self-reported histories of bleeding and hemorrhagic disease, current use of another anticoagulant, severe renal dysfunction, or no access to WeChat or phone; the other 572 patients were evenly divided into two groups. During the 90-day follow-up period, 107 patients in the PEFS group and 84 in the UC group were subsequently excluded due to completion at follow-up prior to the COVID-19 pandemic. Finally, the results of 381 patients were analyzed, including 179 participants from the PEFS groups and 202 participants from the UC group.

### Baseline Characteristics

Patient demographic information and clinical characteristics were described in **Table 1**. The median age in the UC group and PEFS group was 75.2 ± 7.1 and 76.3 ± 7.8, respectively. Both groups were well matched for demographic and clinical characteristics. The difference between the two groups at baseline did not reach statistical significance (P>0.05) (**Table 2**). Notably, most enrolled patients were co-administrated with statins, anti-hypertension agents, β-blockers, antiplatelet, anti-arrhythmic agents, and proton pump inhibitors.

**Table 2:** Baseline demographic and clinical characteristics.

	Usual Care(n=202)	Intervention(n=179)	P value
**Baseline Characteristics**			
Age, years; mean (SD)	75.2(7.1)	76.3(7.8)	0.401
Gender, Male; n (%)	60.4	61.5	0.833
Smoking (%)	22.3	22.3	0.987
Alcohol (%)	15.8	16.2	0.924
BMI, kg/m^2^; mean (SD)	23.2(1.6)	23.2(2.5)	0.092
Rivaroxaban daily dose, mg; mean (SD)	13.9(4.6)	13.8(4.9)	0.463
**Complication**			
Hypertension, (%)	63.9	68.7	0.318
Dyslpidemia, (%)	7.4	4.5	0.227
Diabetes, (%)	16.8	22.9	0.137
Chronic kidney disease, (%)	18.3	21.2	0.476
Stroke/TIA, (%)	18.8	25.1	0.135
Liver Disease , (%)	0	0.6	0.287
Heart Failure, (%)	8.9	4.5	0.086
**Laboratory tests**			
eGFR, mL/(min·1.73 m^2^); mean (SD)	79.7(49.4)	73.5(18.8)	0.068
LDL, mmol/L; mean (SD)	2.0(0.9)	2.0(0.8)	0.571
Hct,%; mean (SD)	41.0(6.2)	40.9(4.6)	0.168
Hb, g/L; mean (SD)	135.3(17.4)	135.2(16.1)	0.525
PLT, 100*10^9^/L; mean (SD)	193.7(58.1)	196.2(60.0)	0.961
ALT, U/L; mean (SD)	23.8(15.8)	25.3(22.2)	0.454
HbA1c, %; mean (SD)	6.1(1.5)	6.1(0.9)	0.150
CK-MB, IU/L; mean (SD)	14.6(7.4)	17.4(17.5)	0.190
NT-proBNP, pmol/L; mean (SD)	1099.3(1452.9)	1340.7(2510.8)	0.142
APTT, s; mean (SD)	28.3(3.8)	28.6(4.0)	0.177
PT, s; mean (SD)	12.6(3.1)	12.7(2.7)	0.635
TT, s; mean (SD)	19.5(12.8)	20.6(17.4)	0.125
D-Dimer, mg/L; mean (SD)	0.9(1.0)	0.8(1.3)	0.311
INR; mean (SD)	1.1(0.32)	1.1(0.25)	0.960
**Co-medication**			
Statin, (%)	53	46.9	0.239
Anti-hypertension agent, (%)	64.9	70.4	0.249
β-blockers, (%)	60.4	52.5	0.121
Antiplatelet, (%)	15.8	10.6	0.135
Anti-arrythmic agent, (%)	35.1	30.2	0.301
PPI, (%)	55.9	56.4	0.924
CHA2DS2-VASc; mean (SD)	3.0(1.3)	3.2(1.4)	0.362
HAS-BLED; mean (SD)	2.0(0.8)	2.0(0.8)	0.251

The data are shown as mean (SD) or %. SD, standard deviation; BMI, body mass index; TIA, transient ischemic attack; eGFR, estimated glomerular filtration rate; LDL, low-density lipoprotein; Hct, hematocrit; Hb, hemoglobin; PLT, platelet; ALT, alanine aminotransferase; CK-MB, creatine kinase-MB; NT-proBNP, N-terminal pronatriuretic peptide; APTT, activated partial thromboplastin time; PT, prothrombin time; TT, thrombin time; INR, international normalized ratio; PPI, proton pump inhibitor.

### Clinical Outcomes Assessments

During the 90-day follow-up period, there was no significant difference between the two groups in terms of the incidence of systemic thromboembolism (including stroke, pulmonary embolism, venous thromboembolism, and cardiac embolism), heart failure (LVEF < 40%), and occurrence ratio of left atrial dilation, which is defined as enlargement of left atrial diameter (LAD) >40 mm (**Table 3**).

**Table 3:** Clinical outcomes in patients between the UC and PEFS groups.

	UC	PEFS	P value
Systemic thromboembolism, (%)	9.4%	7.9%	0.675
Stroke, (%)	2.0%	1.7%	0.825
PE, (%)	1.4%	1.7%	0.881
VTE, (%)	3.0%	1.7%	0.318
Cardiac embolism, (%)	3.0%	2.8%	0.918
LAD > 40mm, (%)	74.8	73.2	0.728
LVEF < 40%, (%)	4.0	2.2	0.336

PE, pulmonary embolism; VTE, venous thromboembolism; LAD, left atrial dimension; LVEF, left ventricular ejection fraction.

Systemic thromboembolism is defined as stroke, PE, VTE, or cardiac embolism.

### Anticoagulation-Related Complications

The frequencies of bleeding events in the UC and PEFS group are shown in **Table 4**. Overall, gastrointestinal bleeding, which is the most common bleeding type, was more frequent in the UC group than the PEFS group (12.4% vs. 6.1%, P = 0.038). Moreover, skin ecchymosis occurred more frequently in the UC group than the PEFS group (4.5% vs. 0.6%, P = 0.018). The cumulative incidences of bleeding complications, such as hematuria, epidural hematoma, operation site hemorrhage, and bleeding gums during anticoagulation therapy, were similar in both groups (P > 0.05). There was no significant difference between the two groups with respect to the levels of Hb, Hct, PLT, and PT (P > 0.05).

**Table 4:** Anticoagulation complications comparison within 90 days.

	UC	PEFS	P value
**Bleeding Complications**			
Gastrointestinal hemorrhage, (%)	12.4	6.1	0.038*
Hematuresis, (%)	3.5	3.9	0.818
Epidural hematoma, (%)	1.0	1.1	0.903
Epistaxis, (%)	1.0	0.6	0.634
Operation site hemorrhage, (%)	2.5	2.2	0.877
Bleeding gums, (%)	2.0	1.7	0.825
Skin ecchymosis, (%)	4.5	0.6	0.018*
**Laboratory parameters**			
Platelet count < 125, (%)	9.9	8.4	0.608
Male: Hb < 120, (%)	10.9	9.5	0.654
Female: Hb < 110, (%)			
PT > 13s, (%)	17.3	12.3	0.169

Hb, hemoglobin; PT, prothrombin time; * represented P < 0.05.

**Figure 2:**
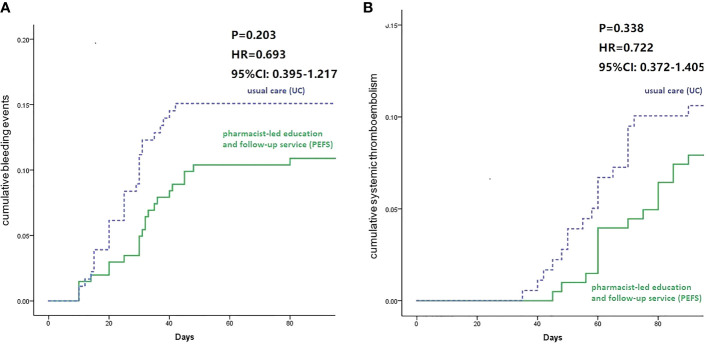
Survival curve of bleeding ratio **(A)** and systemic thrombosis ratio **(B)** for 90-day follow-up between groups.

The cumulative incidence of bleeding events from the Kaplan-Meier estimator was higher in the UC group compared to the PEFS group (13.3% vs. 12.3%), but the difference was not statistically significant (log-rank test, p-value = 0.203). The hazard ratio for UC vs. PEFS groups for the bleeding events was 0.639 (95% CI: 0.395–1.217) (**Figure 2A**). At the 1-year follow-up, the primary rates of systemic thromboembolism occurrence ratio between the UC and PEFS group were 9.4% and 8.9%, respectively. There was no significant difference in thrombosis occurrence ratio after pharmacist intervention when compared with the UC group (P = 0.338, HR: 0.722, 95% CI: 0.372- 1.405), as shown in **Figure 2B**.

### Anticoagulation-Related Interventions

**Figure 3:**
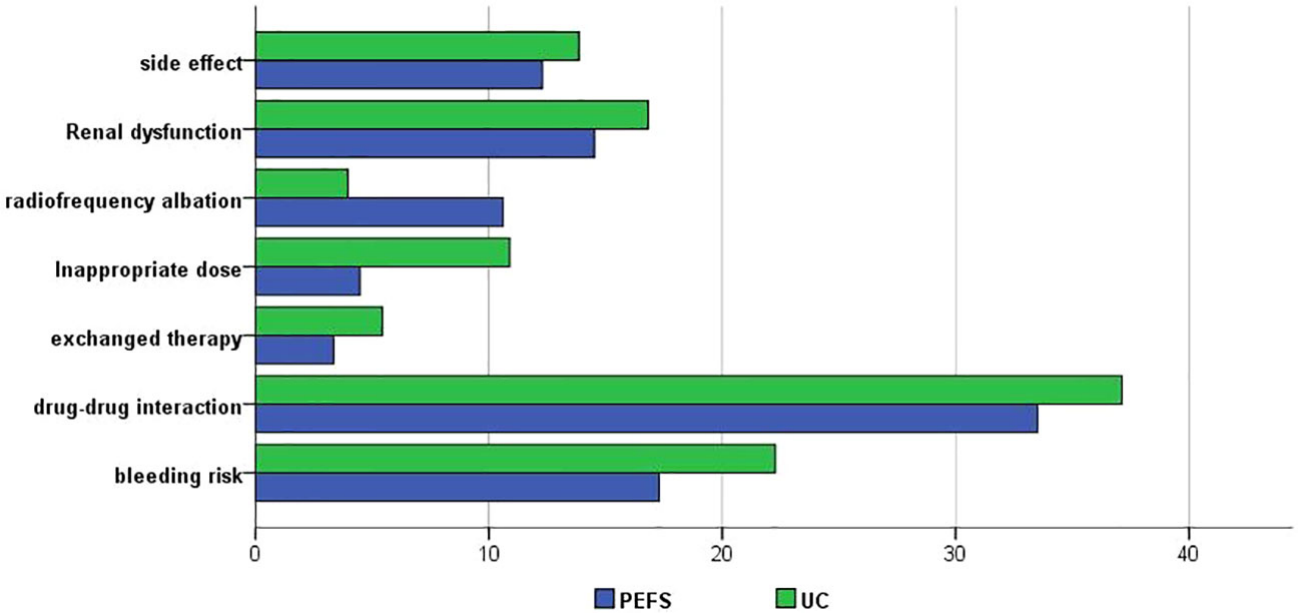
Anticoagulation interventions categorized by provider type.

The overall proportion of patients receiving rivaroxaban therapy was similar in the UC and PEFS groups. The comparison of interventions categorized by provider type between the two groups was shown in **Figure 3**. Compared to the PEFS group, patients in usual care group are more likely to present with anticoagulation therapy related side effects (13.9% vs. 12.3%), higher bleeding risk (22.3% vs. 17.3%), and a high rate of exchanged therapy (5.4% vs. 3.4%). It also worth mentioning that more patients in the PEFS group are willing to accept radiofrequency ablation (10.06% vs. 4%) (**Figure 3**).

### Duration Before the First Outpatient Revisit After Discharge

**Figure 4:**
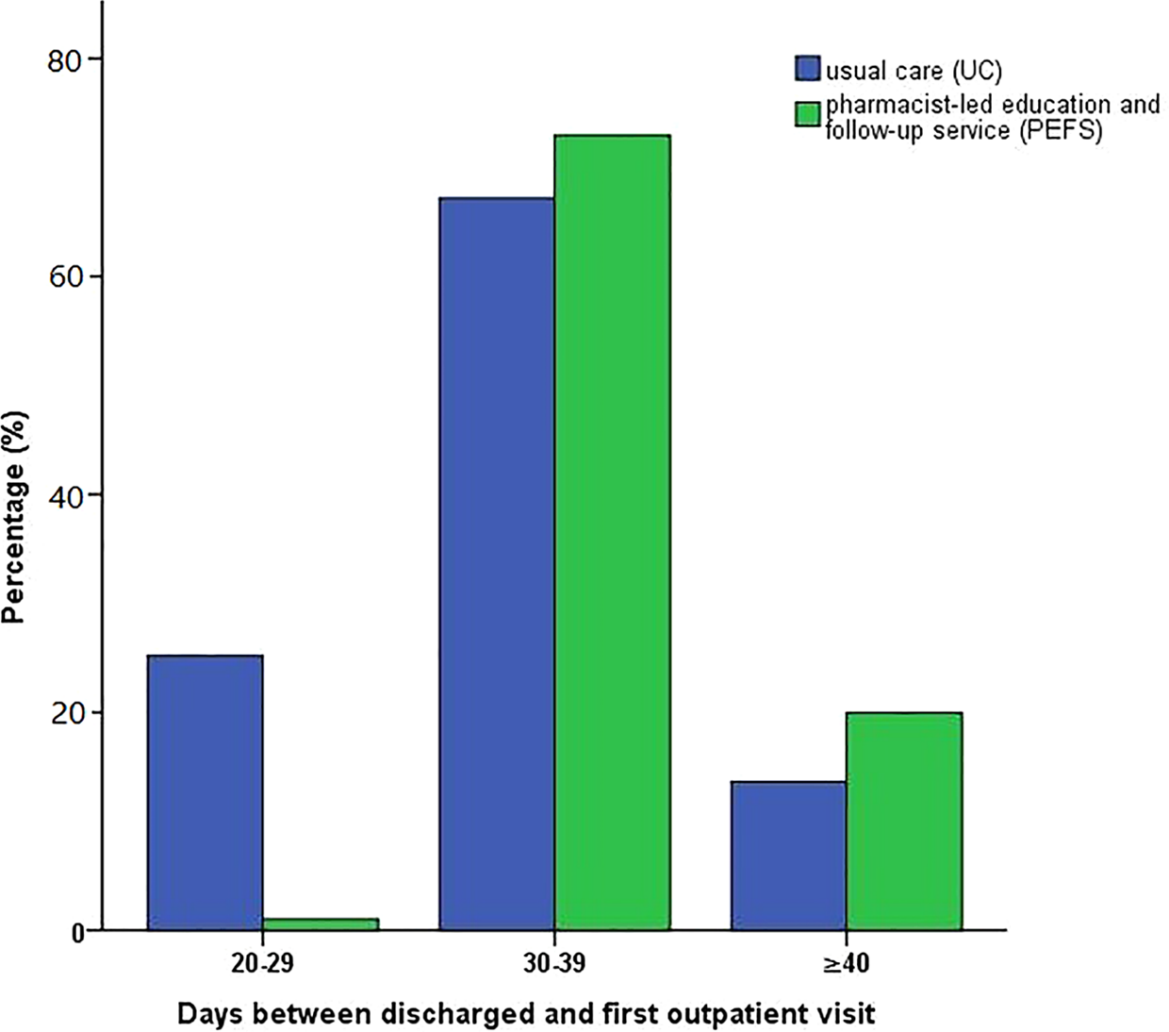
Comparison for the first outpatient visit after discharge.

Within the first 30 days after discharge, the ratio for the first outpatient revisit was higher in the UC group than that of the PEFS group (23.7% vs. 1.1%, P < 0.001). The ratio of patients presenting for the first outpatient visit in the period of 30-39 days post-discharge was higher in the PEFS group compared to the UC group (63.5% vs. 77.6%, P = 0.003*). More patients in the PEFS group had a longer duration (beyond 40 days) before their first outpatient visit after discharge (12.8% vs. 21.3%, P < 0.001) (**Figure 4**).

## Discussion

To our best knowledge, this study is the first to elucidate the effects of remote instruction by pharmacists on the safety and efficacy of rivaroxaban in domiciliary geriatric patients with valvular atrial fibrillation (NVAF). During the epidemic period, in order to reduce cross-infection during hospital visits, pharmacists provide remote pharmaceutical services through WeChat and phone calls for geriatric patients with NVAF medicated on rivaroxaban, such as patient counseling, medication guidance, etc. Our results demonstrate that patients receiving frequent instructive contacts related to their anticoagulation therapy were associated with a reduced rate of anticoagulation bleeding events, which subsequently minimizes the frequency of hospital readmission. As pharmacists are highly accessible primary healthcare providers, remote instruction by pharmacist could have major public health implications in reducing the burden of cardiovascular disease if these practices were widely adopted (Tsuyuki et al., 2016). Our results enriched the reported literature on the impact of clinical pharmacist care on individual components of anticoagulation therapy (Ashjian et al., 2017; DiRenzo et al., 2018). This study indicated that as part of the healthcare provider team, pharmacists are well-positioned to provide motivational advice and patient counseling to promote patient understanding of anticoagulation therapy, patient awareness of potential adverse events, and the importance of medication adherence during the epidemic period.

Meanwhile, poor metabolic function, low body mass index, malnutrition, and other reasons can all increase the risk of bleeding in geriatric patients (Nielsen et al., 2017; Sinigoj et al., 2020). Therefore, we need to strengthen pharmaceutical care for the geriatric population prescribed with rivaroxaban (Conti et al., 2020). In this study, we described the clinical pharmacist intervention on anticoagulation in geriatrics and recorded the patient acceptance rates. Our results demonstrated that the acceptance rate of clinical pharmacist recommendations was nearly 95%. The high acceptance rate indicated that clinical pharmacists now play an important role in medication management and are highly trusted by patients. This might also reflect that clinical pharmacist routine work to make appropriate decisions regarding drug therapy is fully accredited by other clinicians. Our task is to make appropriate clinical decisions in anticoagulation therapy and interact regularly with other health-care professions, thereby contributing to the improvement of outcomes and minimization of adverse drug reaction. Previous studies indicated that most recurrent thrombosis and bleeding events happened in the first month following the adequate anticoagulation (Limone et al., 2013); current American College of Chest Physician guidelines for the treatment and prevention of thrombosis recommend at least 3-months of anticoagulation with rivaroxaban (Linkins et al., 2012). Therefore, we completed a three-month follow-up in this study.

Although several studies have compared outcomes of pharmacist-led anticoagulation services to usual care in recent three years (Aidit et al., 2017; Virdee and Stewart, 2017; Mifsud et al., 2019; Perlman et al., 2019; Liang et al., 2020), prior studies only evaluated the occurrence of thrombosis and bleeding during anticoagulation. The present study tracked the cardiac function by performing transesophageal echocardiograms as a separate category in addition to the occurrence of thrombosis. While previous studies indicated superior anticoagulation management and better patient satisfaction with pharmacist intervention service compared to the doctor-based clinic (Hawes et al., 2018; Roseau et al., 2020), our study indicated no significant differences among all groups in terms of thrombosis occurrence related to the quality of life. One potential explanation might be that unlike the narrow therapeutic range of warfarin, rivaroxaban has a wider therapeutic range by providing frequent anticoagulation *via* inhibiting coagulant factor X when given as a fixed-dose. Also, rivaroxaban acts as a direct oral factor Xa inhibitor, which could prevent thrombosis *via* the intrinsic and extrinsic coagulation pathways (Perzborn et al., 2011).

We were not able to detect a discernable benefit of PEFS care over UC care regarding clinical endpoints in patients receiving rivaroxaban therapy. Left atrial size enlargement and LVEF were predictors of mortality for both cardiovascular issues and all-cause mortality. Our findings suggested that PEFS care could improve cardiac function on LVEF probably due to the anti-inflammatory and anti-fibrotic effects by rivaroxaban (Niessen et al., 2008). Previous reports indicated that rivaroxaban may help to prevent cardiac remodeling by reducing inflammation and fibrosis associated with a decrease in the expression levels of protease activation receptors (PARs) in the left ventricular independent of its anticoagulant effect, and subsequently, improves cardiac function (Goto et al., 2016). Another possible reason might be that pharmacist care included rational use of renin angiotensin aldosterone system (RAAS) inhibitors and beta-blockers appear to prevent new-onset AF in patients with left ventricular dysfunction.

Besides the anti-thrombosis effect, the safety profile such as bleeding complications needed to be taken into account, especially for geriatric patients (Muniz Lobato et al., 2018; Silverio et al., 2019; Kirchhof et al., 2020). There were slightly more bleeding episodes in the UC cohort compared to the PEFS cohort, particularly with hematuria, epistaxis, and operation site hemorrhage events. Patients in the PEFS group tended to receive more frequent contacts related to their anticoagulation therapy from their pharmacy providers than those in the UC group. Besides, many physicians thought these bleeding events are well-tolerated. Therefore, patients may have been at higher bleeding risk. The bleeding occurrence managed by the UC group was higher than those in the PEFS group, including gastrointestinal hemorrhage and skin ecchymosis. A possible explanation for this observation is that PEFS pharmacists were more likely to attend patients (online) and document episodes of bleeding than providers in the UC group.

In the present study, pharmacists were consulted chiefly for drug interactions, followed by questions related to bleeding risk, drug selection in patients with renal insufficiency, other adverse reactions of rivaroxaban, and the need for radiofrequency ablation. One clinical study (Fogg et al., 2012) exploring the focus of drug counseling showed that after the pharmacist-led drug counseling, more than 90% of patients wanted additional information on the effect of the drugs, the mechanism of drugs, and the management when missed dose. 70% to 80% of patients were concerned about the solutions of adverse reactions; 60% to 70% of patients focused on medication course, and 50% to 60% on drug interactions. The focus of patient consultation in this study differs from that of the previous literature. This was due to the fact that in the initial stage of the study, pharmacists had provided the medication instruction of rivaroxaban to the patients, which included the effect, usage, and treatment measures of missed doses of rivaroxaban. In the following remote consultation, patients focused more on the adverse reactions and drug interactions, which may be related to the relatively brief information listed in the guidance. In other words, patients had more demands for individualized guidance on adverse reactions and drug interactions.

The time interval to see a doctor after discharge was significantly delayed in the intervention group compared with the conventional treatment group. One study (Motulsky et al., 2020) demonstrated that 89% of patients reported reduced anxiety and fewer emergency visits after consulting a pharmacist. About 77% of the patients reported no need to visit a hospital within a week after consulting a pharmacist. Similar to previous literature, in the present study, pharmacists established a stable connection with patients through convenient and non-contact ways, such as WeChat and telephone, which postponed the first visit after discharge. At the time of follow-up, pharmacists discovered drug-related problems for patients, educated patients on possible drug interactions of rivaroxaban, optimize combination schemes, determine whether to visit physicians according to the degree and type of bleeding and provided patients with measures to prevent potential bleeding. These pharmaceutical care interventions delayed the time to first hospital visit after discharge.

## Conclusion

Our study demonstrated that the pharmacist-led intervention could reduce the occurrence of bleeding events, especially for gastrointestinal bleeding and skin ecchymosis during anticoagulation with rivaroxaban, thus enhancing safety. Moreover, pharmacist care could postpone the first outpatient visits after discharge compared with the outcomes of patients with usual care.

## Limitation

Some limitations of the present study are as follow: Firstly, the study is a single-site cohort design with a relatively small sample size of 381 participants, which weakened the reliability of the study. In the future, large prospective, randomized, controlled trials are needed to evaluate the influence of long-term online pharmacist intervention on geriatric patients treated with rivaroxaban. Secondly, long-term follow-ups should be taken into consideration in future studies. In addition, although some bleeding events were identified over the telephone, it could not be fully attributed to the adverse drug reaction; an optimized follow-up scheme should be employed in the future. Lastly, we acknowledged that there might be a genetic influence on individual rivaroxaban metabolism that could have affected the outcome of therapy.

## Data Availability Statement

The raw data supporting the conclusions of this article will be made available by the authors, without undue reservation, to any qualified researcher.

## Ethics Statement

This study was conducted in compliance with the Helsinki Declaration and Good Clinical Practice In review and was approved by the Ethics Committee of Zhongshan Hospital. A written informed consent was signed by all participants before the commencement of the clinical studies.

## Author Contributions

XiaoyeL, CZ, and QL conceived the study and wrote the paper. WL, YZ, QX, and XiaoyuL enrolled the patients and collect information. XiaoyuL contributed to the data statistical analysis.

## Funding

This study was supported by the Project of Shanghai Municipal health planning commission (NO. 2016ZB0301).

## Conflict of Interest

The authors declare that the research was conducted in the absence of any commercial or financial relationships that could be construed as a potential conflict of interest.
